# Isolated and Syndromic Genetic Optic Neuropathies: A Review of Genetic and Phenotypic Heterogeneity

**DOI:** 10.3390/ijms26083892

**Published:** 2025-04-20

**Authors:** Marco Zeppieri, Caterina Gagliano, Marco Di Maita, Alessandro Avitabile, Giuseppe Gagliano, Edoardo Dammino, Daniele Tognetto, Maria Francesca Cordeiro, Fabiana D’Esposito

**Affiliations:** 1 Department of Ophthalmology, University Hospital of Udine, 33100 Udine, Italy; 2 Department of Medicine, Surgery and Health Sciences, University of Trieste, 34127 Trieste, Italy; tognetto@units.it; 3 Department of Medicine and Surgery, “Kore” University of Enna, Piazza dell’Università, 94100 Enna, Italy; caterina.gagliano@unikore.it; 4 Mediterranean Foundation “G.B. Morgagni”, Via Sant’Euplio, 95100 Catania, Italy; mdimaita86@gmail.com; 5 Eye Clinic Catania University San Marco Hospital, Viale Carlo Azeglio Ciampi, 95121 Catania, Italy; alessandro.avitabile2001@gmail.com (A.A.); giuseppe.gagliano99@gmail.com (G.G.); edoardo.damiano@gmail.com (E.D.); 6 Imperial College Ophthalmic Research Group (ICORG) Unit, Imperial College, 153-173 Marylebone Rd, London NW1 5QH, UK; m.cordeiro@imperial.ac.uk (M.F.C.); f.desposito@imperial.ac.uk (F.D.); 7 Department of Neurosciences, Reproductive Sciences and Dentistry, University of Naples Federico II, Via Pansini 5, 80131 Napoli, Italy

**Keywords:** hereditary optic neuropathy, Leber hereditary optic neuropathy (LHON), dominant optic atrophy (DOA), wolfram syndrome, mitochondrial DNA, optic atrophy, gene therapy

## Abstract

Nonsyndromic and syndromic hereditary optic neuropathies (HONs) encompass a variety of genetic illnesses that cause progressive optic nerve damage, resulting in considerable vision impairment. These disorders result from pathogenic variants in mitochondrial or nuclear DNA, impacting essential cellular processes like oxidative phosphorylation, mitochondrial dynamics, and neuroprotection. Advances in next-generation sequencing (NGS) have significantly improved the identification of genetic variations, enabling precise diagnoses and genotype–phenotype correlations. This review consolidates current knowledge regarding the classification, molecular pathogenesis, clinical manifestations, diagnostic methodologies, and emerging therapeutic strategies for HONs. The critical role of mitochondrial dysfunction in optic nerve degeneration highlights the necessity for multimodal therapeutic approaches. Recent clinical trials evaluating gene therapy for Leber hereditary optic neuropathy (LHON) and neuroprotective strategies in dominant optic atrophy (DOA) are discussed. Additionally, individualized therapeutic interventions, as demonstrated by recent case studies involving tailored gene therapies, are evaluated. The integration of molecular and imaging biomarkers in future personalized treatment strategies aims to enhance prognosis and therapeutic outcomes.

## 1. Introduction

Hereditary optic neuropathies (HONs) are a genetically heterogeneous group of disorders that are primarily defined by progressive optic nerve dysfunction with ensuing severe visual impairment or blindness [1]. The optic nerve conveys visual stimuli from the retina to the brain through nerve fibers that originate from the retinal ganglion cells. Optic nerve fibers are extremely delicate and require high energetic support, which is provided by intense mitochondrial activity [1,2]. Optic neuropathies can be caused by several different causes, such as congenital abnormalities, metabolic impairment, glaucoma, infections, inflammation and immune-mediated disorders, trauma, compressive disorders, and genetic factors [3].

Hereditary optic nerve disorders usually have insidious onset and progression; therefore, diagnosis is often delayed or incorrect. Inherited optic neuropathies have a poor visual prognosis and significantly decrease the quality of life of affected patients and their relatives [4,5]. Diagnosis and management of patients with optic neuropathies are based on an accurate neuro-ophthalmological examination, including tonometry, gonioscopy, and fundus examination, in association with several tests, such as automated visual field test, optical coherence tomography (OCT); electrophysiological studies, including visual evoked potentials (VEP); and electroretinography (ERG) [6].

Clinical fundus examination usually demonstrates bilateral optic disc pallor. Gonioscopy and tonometry are necessary to exclude acquired causes of optic neuropathies such as open-angle and angle-closure glaucoma. OCT is a noninvasive imaging technology that provides a high-quality cross-sectional image of the retinal ganglion cells and retinal nerve fiber layers, which is useful for diagnosing and surveilling optic nerve disorders [7]. Visual evoked potentials estimate the electrical signal generated in the brain in response to visual stimulation. VEPs quantify the functional integrity of the optic nerve pathway from the eye to the cerebral visual cortex. ERG measures electrical activity generated directly by retinal neurons [8].

A wide spectrum of genetic alterations causes inherited optic neuropathies. Some entities, such as dominant optic atrophy (DOA) and Leber hereditary optic neuropathy (LHON), are defined as isolated optic neuropathies because they are related to definite pathogenic variants in mitochondrial or nuclear DNA that exclusively affect visual function without other systemic abnormalities. On the other hand, syndromic optic neuropathies are caused by mutations with a pleiotropic effect and are associated with a wide spectrum of abnormalities affecting other organs in the body [6]. Advances in next-generation sequencing (NGS) and expanded molecular testing have revealed that a broad array of mitochondrial and nuclear genes can underlie either isolated (nonsyndromic) optic nerve disease or complex multisystem disorders (syndromic forms) [9]. NGS and genome-wide association studies (GWASs) have greatly enhanced our understanding of the molecular causes of hereditary optic neuropathies [10].

Pure hereditary optic neuropathies are characterized by visual impairment without associated signs, while syndromic forms, such as Wolfram syndrome or Friedreich’s ataxia, display other features, such as diabetes mellitus, hearing loss, and neurological manifestations [11,12]. Despite such clinical variability, one common thread running through all these disorders is the increased vulnerability of retinal ganglion cells (RGCs) to mitochondrial injury and oxidative stress. The current review tries to integrate available knowledge on the molecular etiology and phenotypic manifestation of isolated and syndromic hereditary optic neuropathies and review the new diagnostic and therapeutic possibilities with promise for improving patient care.

The present review will focus on purely genetic primary optic nerve degenerations. Complex traits such as glaucomatous optic neuropathy or optic neuropathy related to various types of anterior segment dysgenesis [13] are reviewed elsewhere.

## 2. Methods

In January 2025, an extensive search was performed throughout multiple esteemed databases, including PubMed, Medline, the Cochrane Library, and ClinicalTrials.gov, adhering to the PRISMA (Preferred Reporting Items for Systematic Reviews and Meta-Analyses) principles to guarantee transparency and rigor. The search method utilized Medical Subject Headings (MeSH) terminology and keywords pertinent to this study’s emphasis, such as “genetic optic neuropathies”, “molecular variants”, “Leber’s hereditary optic neuropathy (LHON)”, and “dominant optic atrophy (DOA)”. Only publications in the English language were included, with no limitations on publication date or status.

The inclusion criteria consisted of peer-reviewed studies that examined the molecular genetics of ocular neuropathies. Research of interest encompassed retrospective studies, prospective studies, randomized clinical trials, and case series that offered comprehensive genetic data or insights regarding molecular variants and pathogenic mutations linked to optic neuropathies. Articles including human participants of any age or gender were deemed appropriate for inclusion.

Exclusion criteria were based on studies that showed insufficient or incomplete data on genetic determinants, non-peer-reviewed status, or a focus on subjects unrelated to the genetic basis of ocular neuropathies. Furthermore, studies lacking human subjects or published in languages other than English were omitted.

Data were separately extracted by two reviewers who assessed titles, abstracts, and full-text publications for eligibility, resolving inconsistencies by discussion and consensus. The online Mendelian Inheritance in Man (OMIM) site was utilized to obtain the latest genetic information regarding genes linked to ocular neuropathies. The systematic review rigorously followed the PRISMA standards, meticulously recording each phase of the search, selection, and data extraction procedure, thus improving the transparency, accuracy, and repeatability of the results.

## 3. Results

### 3.1. Molecular Causes of Optic Neuropathies

This section provides a comprehensive analysis of genetic optic neuropathies, focusing on genetic alterations, clinical manifestations, genotype–phenotype correlations, and treatment strategies.

The main isolated forms are described. Some of the reported genes can also cause more complex phenotypes with additional features, determining syndromic phenotypes.

#### 3.1.1. Isolated (Nonsyndromic) Genetic Optic Neuropathies

##### Leber Hereditary Optic Neuropathy (LHON)

Leber hereditary optic neuropathy (LHON) is the most frequent mitochondrial disease [14]. Its prevalence ranges from 1 in 27,000 to 1 in 45,000 in Europe [15,16,17]. The disease results from mitochondrial DNA (mtDNA) mutations and follows maternal inheritance. It affects young men in 80–90% of cases and usually leads to optic atrophy within 1 year of disease onset [14]. In 95% of cases, LHON is determined by point mutations in the mtDNA genes encoding proteins of mitochondrial complex I (NADH: ubiquinone oxidoreductase) [18]. Retinal ganglion cells (RGCs) are particularly vulnerable to mitochondrial dysfunction caused by these mutations [8]. Patients typically start experiencing painless, subacute central vision loss in one eye around the second or third decade of life. The second eye is usually affected within 8 weeks [18]. Macular RGCs giving origin to the papillomacular bundle are preferentially affected, resulting in color vision impairment, central scotomas, and temporal pallor of the optic disc [17].

Loss of macular RGCs precedes the clinical onset of symptoms [19]. Traditionally, LHON patients’ phenotypes can be divided into three categories: asymptomatic mutation carriers, acute LHON (disease duration of 1 year or less), and chronic LHON (disease duration of more than 1 year) [20]. According to the latest consensus on LHON, there are four clinical stages of LHON, based on the time from onset and the results of clinical investigations: asymptomatic (mutation carriers), subacute (<6 months from onset), dynamic (6–12 months), and chronic (>12 months) [14]. Other three clinical variants can be considered: slowly progressive, childhood disease (onset before the age of 12 years), and late onset (onset after 45 years) [14]. Asymptomatic carriers of LHON mutation may present typical signs on fundus examination and OCT, such as optic disc hyperemia, vascular tortuosity, peripapillary telangiectasias, and increased thickness (swelling) of the peripapillary retinal nerve fiber layer (pseudoedema). However, in some cases, fundus examination can be unremarkable [17]. Loss of macular RGCs at OCT, with subsequent deterioration of visual acuity and visual field, characterizes the symptomatic stage of LHON. Thinning of the macular RGC layer is usually completed within 6 months [19]. Retinal nerve fiber layer (RNFL) thickness increases in the first 6 months, with a following thinning in specific quadrants [20]. In the first weeks, disease progression can be acute or subacute. Within the first 2 months, best corrected visual acuity (BCVA) stabilizes, but OCT and visual field findings may continue to evolve, reaching a plateau around 1 year after onset [19]. Hence, the chronic phase of the disease starts. In the chronic atrophic phase, optic nerves appear pale, and vision is profoundly impaired [14]. Vision can recover to some extent depending on the age of onset and the mutation subtype [21].

Diagnosis of LHON is primarily clinical. In a young man with subacute vision loss, a maternal family history of vision loss, central scotoma, dyschromatopsia, and optic nerve pallor, LHON should be highly suspected [14]. Definitive diagnosis of LHON is achieved by the molecular identification of one of the three mtDNA mutations (m.11778G.A/MT-ND4, m.3460G.A/MT-ND1, m.14484T.C/MT-ND6), which account for about 90% of cases [14,22].

Sequencing the entire mtDNA is advisable to find rare mtDNA variants if the first genetic test is negative, but there is a high index of clinical suspicion [23]. Penetrance of LHON is incomplete and variable. Several environmental and epigenetic factors can influence disease expression [24]. The phenotype of patients carrying the pathogenic variants of LHON is variable between sex and individuals: around 90% of females and 50% of males carrying the variant are unaffected. Younger age and the mutation 14484/ND6 are positive prognostic factors [14]. Different mechanisms, such as heteroplasmy, epigenetic factors, and hormonal factors, can explain this phenomenon [25]. Pathogenic variants in the *DNAJC30*, *MACT*, *MECR*, and *NDUFS2 genes* have been recently described as related to recessive forms of LHON [25].

Idebenone, a short-chain benzoquinone, is the only available approved drug for LHON. It was approved by EMA in June 2015 for individuals older than 12 years with LHON [24]. Idebenone has proven to be effective in stabilizing and improving vision in patients treated within the first year of onset [24]. It is indicated for the treatment of adolescents and adults affected by LHON at a daily dose of 900 mg [26,27,28]. It helps mitochondria bypass complex I and preserve ATP production in RGC. Idebenone should be administered as soon as possible at 900 mg/day within the first year of disease onset, while there is no proven evidence of its efficacy in the chronic phase. Treatment should continue for at least 1 year to evaluate therapeutic response. Best corrected visual acuity and automated visual field tests should be used to monitor disease progression [14].

The m. 1178G>A variant is the most common mutation and accounts for almost 70% of cases in America and Europe. This variant causes a severe form of LHON [29]. The REFLECT trial, a phase 3 randomized, double-masked, placebo-controlled trial, investigated the efficacy and safety of bilateral intravitreal injection of lenadogene nolparvovec using a recombinant adeno-associated virus vector in LHON patients with the m. 1178G>A mutation. Authors of the REFLECT trial demonstrated a statistically significant improvement in BCVA from baseline to 1.5 years in bilaterally and unilaterally treated patients in comparison with placebo [29].

##### Dominant Optic Atrophy (DOA)

Dominant optic atrophy (DOA, OMIM 165500) is the most common Mendelian inherited optic neuropathy [6]. It is transmitted as an autosomal dominant trait. It is characterized by bilateral degeneration of the retinal ganglion cells (RGCs) and their axons, eventually leading to optic nerve atrophy and visual loss [13]. Its prevalence ranges from 1/50,000 to 1/10,000 [6]. Clinical features are determined by the loss of RGCs in the papillomacular bundle [30]. Fundus examination in DOA patients demonstrates temporal pallor of the optic discs with atrophic rim, while OCT imaging shows reduced retinal nerve fiber layer thickness in the papillomacular bundle and thinning of the ganglion cell layer [31,32]. The disease is characterized by paracentral or central visual field scotomas and generalized dyschromatopsia or tritanopia, an uncommon form of “blue-yellow” color blindness [30].

Visual evoked potentials are usually absent or delayed, while pattern electroretinogram reveals an abnormal N95:P50 ratio, with a reduction in the N95 waveform [30]. Patients typically complain of an insidious, progressive bilateral vision loss starting in childhood, but it can also manifest during adulthood. Vision impairment usually progresses during puberty until adulthood, with subsequent chronic slow progression [33]. About half of DOA patients are registered as legally blind and are not able to drive [4]. The expressivity of the disease is variable in the same family and between different families. Indeed, visual acuity can range from normal vision to hand movements from a short distance. This phenomenon is related to a combination of epigenetic and environmental influences [13].

Dominant optic atrophy can be divided into syndromic and nonsyndromic forms. Syndromic DOA, also called DOA plus, presents a more severe ocular phenotype and 100% penetrance, while nonsyndromic DOA penetrance is around 70% [34]. There is a significant difference in frequency and type of mutations between nonsyndromic DOA and syndromic DOA: deletions are the most common in the isolated DOA group, while missense variants are the most common in the DOA plus group [34]. Isolated DOA is more likely associated with *OPA1* gene pathogenic variants in exons 8 and 9, while DOA plus is more likely to have pathogenic variants in exons 14, 15, and 17 [34]. Furthermore, DOA plus manifestations are more frequent in patients with maternally inherited *OPA1* variants than in those with paternally inherited variants [34]. Syndromic DOA accounts for 20% of total cases and is characterized by extraocular findings such as cerebellar ataxia, myopathy, peripheral neuropathy, neurosensorial hearing loss, and progressive ophthalmoplegia [35,36].

**Figure 1:**
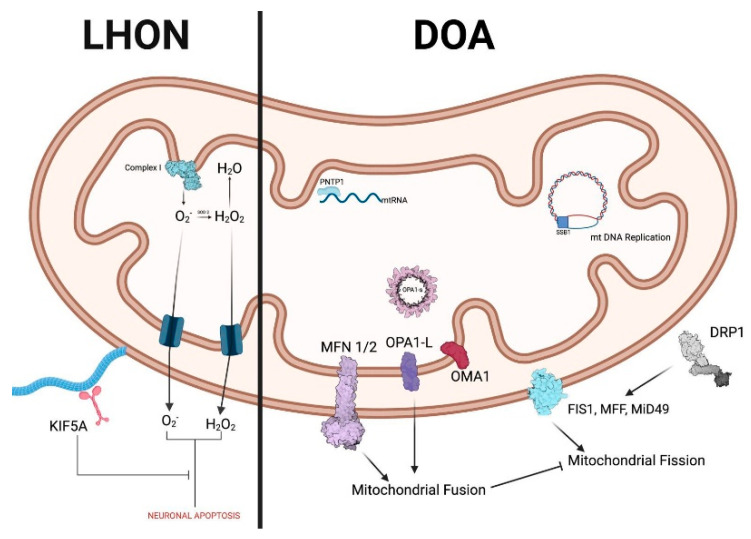
Summary of implicated proteins in LHON and DOA. Created in BioRender. Avitabile, A. (2025) https://BioRender.com/k6on15w (accessed on 6 September 2025).

DOA is a mitochondriopathy, as the related genes encode proteins that are ubiquitously expressed and incorporated into the inner mitochondrial membrane (Figure 1) [37]. An increasing number of nuclear gene variants are being discovered as related to DOA phenotypes, with a dominant but also X-linked or recessive mode of inheritance [10,38] (Table 1). However, pathogenic variants in the autosomal gene *OPA1* account for 70–90% of cases of DOA [39,40,41,42,43]. The *OPA1* gene includes 30 exons, spanning > 90 kbp of genomic DNA [44], and encodes a GTPase protein located in the inner mitochondrial membrane. It regulates several mitochondrial functions, such as mitophagy, stabilization of the respiratory chain complexes, and mitochondrial fusion [45].

More than 500 variants in the *OPA1* gene have been related to pathogenic conditions. Around one-third of these are missense, and around 25% cause aberrant splicing [46]. Most of these variants, such as the commonest c.2708_2711del, determine a premature interruption of translation, causing reduced concentrations of the OPA1 protein. Indeed, haploinsufficiency, which means that a normal copy of the protein is insufficient to maintain its normal cellular function, is the primary molecular mechanism in DOA. Hence, the protein is not expressed, causing mitochondrial failure in retinal ganglion cells, which are highly susceptible to ATP deficiency [47]. Other *OPA1* variants, predominantly missense (such as c.2356-1G>T), cause the so-called dominant-negative (DN) effect, where the impaired protein hinders the function of the wild-type protein [47].

Missense variants are associated with more severe phenotypes and extraocular features [36]. Specific impairment of RGCs, especially those giving origin to the papillomacular bundle, is the responsible pathogenetic mechanism of DOA. The *OPA1* gene is highly expressed in the brain, muscles, and retina. However, RGSs appear to be extremely vulnerable to mutations of *OPA1* and consequent mitochondrial dysfunction [8]. This is probably because the axons of RGCs are very long, connecting the inner surface of the retina to the lateral geniculate nucleus [48]. The intraocular RCG axons are unmyelinated to facilitate light penetration into the retina. Hence, these fibers cannot transmit action potentials through saltatory conduction, consume a large amount of ATP, and have high metabolic requirements; RGCs of the papillomacular bundle are extremely vulnerable due to light-induced oxidative stress and limited blood supply [49,50,51].

Unfortunately, there is no available treatment for dominant optic atrophy, and patients can only benefit from low-vision aids. Patients are advised to avoid tobacco, alcohol, and every medication that may hinder mitochondrial function [48]. Treatment strategies can be classified into three groups: gene therapy, cell-based regenerative therapy, and pharmacological neuroprotection [31]. Idebenone, a synthetic analog of coenzyme Q10 approved for LHON, has been studied as a neuroprotector in patients with DOA. In 2023, Valentin et al. demonstrated that the daily intake of 900 mg of idebenone led to the maintenance of visual function and a significant improvement in vision-related quality of life [52]. However, clinical trials are currently ongoing, and idebenone is not approved for DOA by agencies for drug regulation.

Gene replacement therapy has been tested in mouse models. Sarzi et al. demonstrated that intravitreal injections of an adeno-associated virus vector carrying human OPA1 cDNA into a knockdown OPA1 mice model determined some protection of RGCs from degeneration. Still, overexpression of *OPA1* proved to be deleterious [53]. Research groups are working on developing antisense oligonucleotide (ASO) and small interfering RNAs for DOA [54]. A study by Sladen et al. demonstrated that CRISPR-Cas9 gene editing determined an improvement in DOA phenotype in RGCs derived from patients affected by dominant optic atrophy [55].

##### Other HONs

HONs can also be caused by pathogenic variants in other genes, as summarized in Table 1. Optic neuropathy may be a sole feature or be accompanied by specific ocular and/or systemic involvement [9]. These presentations are relatively rare, highlighting the significance of comprehensive genetic testing in atypical presentations.

**Table 1:** Genes related to isolated/complex phenotypes HONs (nuclear genes) [13,56].

Locus (Phenotype MIM No.)	Gene	Inheritance	Phenotype(s)
OPA1 (165500)	*OPA1*	AD	Optic atrophy Optic atrophy plus
OPA3 (165300)	*OPA3*	AD/AR	Optic atrophy + possible cataract
OPA5 (610708)	*DNM1L*	AD	Optic atrophy
OPA7 (612988)	*TMEM126A*	AR	Optic atrophy + possible HI, CD, ND
OPA9 (616289)	*ACO2*	AD/AR	Optic atrophy + possible ND
OPA10 (616732)	*RTN4IP1*	AR	Optic atrophy + possible ND
OPA11 (617302)	*YME1L1*	AR	Optic atrophy + ND
OPA12 (618977)	*AFG3L2*	AD	Optic atrophy + possible ND
OPA13 (165510)	*SSBP1*	AD	Optic atrophy and retinal abnormalities + possible HI, PN
OPA14 (620550)	*MIEF1*	AD	Optic atrophy
OPA15 (620583)	*MCAT*	AR	Optic atrophy
OPA16 (620629)	*MECR*	AR	Optic atrophy + HI

Legend. MIM: Mendelian Inheritance in Man; AD: autosomal dominant; AR: autosomal recessive; HI: hearing impairment; CD: cardiac defects; ND: neurological disorders; PN: progressive nephropathy.

### 3.2. Syndromic HONs

Given that they result from mitochondrial function impairment, HONs can be part of a more complex systemic spectrum of features and, therefore, various syndromes. Among those, the most frequently encountered are described here.

Wolfram syndrome has an early onset (usually in the first decade) and is characterized by optic atrophy, diabetes mellitus or insipidus, neurosensorial hearing loss, and various neurological impairments [57]. The underlying gene is *WFS1* (OMIM #606201), encoding a ubiquitously expressed protein that localizes primarily in the endoplasmic reticulum (ER) [58]. Although *WFS1* pathogenic variants related to the classic syndromic phenotype are inherited in recessive mode, dominantly acting variants can determine so-called “Wolfram-like” phenotypes, characterized by low-frequency hearing impairment, diabetes mellitus, and/or optic atrophy [59,60,61]. Wolfram syndrome is genetically heterogeneous. Wolfram syndrome-2 (WFS2) is characterized by diabetes mellitus, sensorineural hearing impairment, optic atrophy, neurological signs, and defective platelet aggregation [62]. The underlying defective gene is *CISD2* [63,64,65], inherited recessively.

Friedreich’s ataxia (FRDA) is a severe neurodegenerative autosomal recessive disorder. Optic neuropathy is part of its phenotype in about 25% of affected patients [66], and eye movement abnormalities are present in 91% of patients [67]. Prevalence is about 0.50:100,000, with a carrier rate of 1/60 to 1/100 [68]. The underlying gene is *FXN*, encoding frataxin, a mitochondrial iron chaperone involved in iron–sulfur biogenesis and heme biosynthesis [69]. About 96% of cases are determined by biallelic GAA trinucleotide to repeat expansion in intron 1 of the *FXN* gene. In comparison, approximately 4% are compound heterozygotes for a GAA repeat expansion and an intragenic pathogenic variant or a large deletion [70].

Rojas et al. described the progression of ocular involvement in FRDA. The retinal nerve fiber layer, which is rich in mitochondria, is among the first structures affected. As the disease advances, further degeneration occurs, including damage to the ganglion cell complex (GCC), macular thinning, and optic radiation deterioration [71].

Optic neuropathy has also been described in a small percentage of individuals affected by Charcot–Marie–Tooth disease (CMT), a genetically and phenotypically heterogeneous group of conditions characterized by chronic motor and sensory polyneuropathy [72].

Occasionally, optic neuropathy can also be a pathologic trait of Refsum disease, an autosomal recessive inborn error of lipid metabolism characterized by rod-cone retinal dystrophy, peripheral neuropathy, cerebellar ataxia, and elevated protein levels in the cerebrospinal fluid (CSF). Genetically, Refsum diseases are related to the presence of biallelic variants in the *PHYH* (phytanoyl-CoA hydroxylase) gene, with a consequent failure to metabolize phytanic acid correctly [73].

## 4. Discussion

Hereditary optic neuropathies (HONs), whether syndromic or isolated, are unified by a common molecular theme: retinal ganglion cell (RGC) degeneration driven largely by mitochondrial dysfunction. RGCs have exceptionally high energetic demands and depend on efficient mitochondrial oxidative phosphorylation in their long axons [1,2,9]. Failures in mitochondrial ATP production and excessive reactive oxygen species (ROS) generation are therefore key upstream events that precipitate RGC loss in these disorders [1,2,9]. Both Leber hereditary optic neuropathy (LHON) and dominant optic atrophy (DOA) exemplify this principle—in LHON, primary mutations in mtDNA-encoded respiratory chain subunits (usually complex I) lead to impaired electron transport, energy deficit, and ROS accumulation in RGCs [1,2,22,33]. In DOA, haploinsufficiency or dysfunction of *OPA1*, a nuclear gene encoding a mitochondrial inner membrane GTPase, disrupts mitochondrial fusion and cristae maintenance, compromising respiratory efficiency in RGCs [30,34,35,39,40,45]. The downstream result in both cases is an energy crisis and susceptibility to apoptotic cell death in the optic nerve [9,30,37]. Indeed, *OPA1*-mutant cells show increased mitochondrial fragmentation and a lowered threshold for permeability transition pore opening, linking *OPA1* defects to intrinsic apoptotic pathways in RGCs [37,45]. These mechanistic insights underscore that mitochondrial dysfunction is a central pathogenic driver across HONs [1,2,9], explaining why energetically active RGCs are the first neurons to degenerate in these conditions.

Genotype–phenotype variability in HONs highlights the influence of additional molecular modifiers on this core mitochondrial pathway. In LHON, the same mtDNA mutation can produce widely varying clinical severity and penetrance even within one family [74]. One major factor is heteroplasmy—the proportion of mutant mitochondria—which must typically exceed a threshold in RGCs to cause disease [75]. However, heteroplasmy alone does not explain the clinical variability. Background differences in the mitochondrial genome (haplogroups) and nuclear genome can modulate the phenotype. For example, the mtDNA haplogroup J is known to increase the risk of visual loss in European LHON families carrying the common m. 11778G>A mutation. Such haplotype-specific variants likely alter mitochondrial bioenergetics or ROS handling, acting as genetic modifiers of LHON expression [76]. Additionally, newly identified nuclear gene defects can influence the LHON phenotype—notably, a recent study found biallelic mutations in *DNAJC30* (a complex I assembly factor) that cause an LHON-like optic neuropathy with a recessive inheritance, illustrating how nuclear genome variation can produce an LHON phenotype by impairing mitochondrial complex I repair [77]. Epigenetic regulation and gene–environment interactions further contribute to variability. Epigenomic differences may affect the expression of key mitochondrial proteins or stress response genes in RGCs. Hereditary optic neuropathies (HONs), whether syndromic or isolated, are unified by a common molecular theme: retinal ganglion cell (RGC) degeneration driven largely by mitochondrial dysfunction. RGCs have exceptionally high energetic demands and depend on efficient mitochondrial oxidative phosphorylation in their long axons [1,2,9]. Failures in mitochondrial ATP production and excessive reactive oxygen species (ROS) generation are therefore key upstream events that precipitate RGC loss in these disorders [1,2,9]. Both Leber hereditary optic neuropathy (LHON) and dominant optic atrophy (DOA) exemplify this principle—in LHON, primary mutations in mtDNA-encoded respiratory chain subunits (usually complex I) lead to impaired electron transport, energy deficit, and ROS accumulation in RGCs [1,2,22,33]. In DOA, haploinsufficiency or dysfunction of *OPA1*, a nuclear gene encoding a mitochondrial inner membrane GTPase, disrupts mitochondrial fusion and cristae maintenance, compromising respiratory efficiency in RGCs [30,34,35,39,40,45]. The downstream result in both cases is an energy crisis and susceptibility to apoptotic cell death in the optic nerve [9,30,37]. Indeed, *OPA1*-mutant cells show increased mitochondrial fragmentation and a lowered threshold for permeability transition pore opening, linking *OPA1* defects to intrinsic apoptotic pathways in RGCs [37,45]. These mechanistic insights underscore that mitochondrial dysfunction is a central pathogenic driver across HONs [1,2,9], explaining why energetically active RGCs are the first neurons to degenerate in these conditions.

Syndromic vs. nonsyndromic HONs can be distinguished by the breadth of their molecular impact beyond the optic nerve. Nonsyndromic HONs such as LHON and isolated DOA are largely restricted to RGC degeneration, and their molecular pathology centers on mitochondrial dysfunction within the optic nerve [1,2,9,13]. In these cases, the causative mutations (mtDNA complex I genes in LHON or *OPA1* and a few other optic atrophy genes in DOA) primarily affect cellular energy supply and survival specifically in RGCs [1,2,9,13,33,35]. In contrast, syndromic HONs involve optic neuropathy as part of a wider multisystem disorder, reflecting more ubiquitous cellular defects. For example, Wolfram syndrome (DIDMOAD) is caused by mutations in *WFS1*, a gene encoding an endoplasmic reticulum (ER) protein rather than a mitochondrial protein. The mechanistic consequence is chronic ER stress and impaired calcium homeostasis, leading to apoptosis in pancreatic β-cells and neurons (including RGCs) [11,57,58]. While mitochondrial dysfunction is secondary in Wolfram syndrome (it can arise from ER stress-induced mitochondrial calcium dysregulation), the primary molecular insult lies outside the mitochondria [11,58,60]. Similarly, Friedreich’s ataxia involves optic atrophy alongside spinocerebellar degeneration due to a GAA trinucleotide repeat expansion in the nuclear *FXN* gene. This causes loss of frataxin, a mitochondrial matrix protein critical for iron–sulfur cluster assembly, which in turn leads to iron accumulation and oxidative damage in many tissues [12,66,69,70]. The optic nerves in Friedreich’s ataxia suffer from defective mitochondrial metabolism akin to LHON, but patients also develop cardiomyopathy and ataxia due to frataxin deficiency in those tissues [12,66,71]. Another syndromic example is Refsum disease, an inborn error of peroxisomal phytanic acid metabolism: though not a primary mitochondrial disorder, the toxic buildup of phytanic acid can damage both the retina and the peripheral nerves, including the optic pathways [73]. Thus, syndromic HONs implicate diverse cellular organelles and pathways (ER, nucleus, peroxisome, etc.), and the presence of systemic features indicates a broader molecular perturbation beyond the optic nerve. Interestingly, the distinction between syndromic and nonsyndromic forms is not absolute at the molecular level. Some classically “isolated” optic neuropathy genes have more pleiotropic effects in certain contexts—for instance, about 20% of patients with *OPA1* mutations develop extra-ocular neurological features (hearing loss, myopathy, peripheral neuropathy), a phenotype known as “DOA+” [13,36]. Typically, these cases are due to particular *OPA1* mutations (such as missense changes in the GTPase domain or large deletions) that cause a more profound mitochondrial dysfunction affecting multiple cell types [34,36,37]. In summary, nonsyndromic HONs mainly reflect localized mitochondrial failure in RGCs, whereas syndromic HONs result from more globally acting genetic defects that, while often still impacting mitochondria, manifest in multiple organs. This dichotomy underscores how the molecular scope of the causative mutation—from a single cellular process to multi-organ networks—determines whether an optic neuropathy stands alone or as part of a syndrome [1,2,13,36]. Understanding these differences at the molecular level can inform why some HON patients need purely neuro-ophthalmic management while others require multidisciplinary care (e.g., addressing diabetes and deafness in Wolfram syndrome or cardiac issues in Friedreich’s) [12,57,66], even as research focuses on the shared mitochondrial vulnerability of RGCs across all HONs.

Mechanistic insights have been greatly advanced by animal and cellular models of HONs, which recapitulate disease pathways and enable experimental intervention. In DOA, mouse models haploinsufficient for *Opa1* (the murine *Opa1* gene) develop progressive optic nerve degeneration, mirroring human DOA and validating that a 50% reduction in *OPA1* is pathogenic to RGCs [35,53]. These mice exhibit RGC loss and visual deficits, implicating impaired mitochondrial fusion and disrupted cristae structure as the cause of neurodegeneration [30,53]. Importantly, such models allow the testing of therapies: in one study, an AAV-mediated *OPA1* gene supplementation in an *Opa1*⁺/⁻ mouse effectively preserved RGCs [53], directly linking the molecular defect to a treatable mechanism. Cellular models have provided complementary insights. Patient-derived induced pluripotent stem cell (iPSC) models of DOA have been differentiated into RGC-like neurons, revealing abnormal mitochondrial morphology and impaired bioenergetics, which could be rescued by introducing wild-type OPA1 or stabilizing mitochondrial membranes [30,37]. In LHON, cybrid cell lines (which carry patient mitochondria in a standardized nuclear background) have demonstrated the deleterious effects of specific mtDNA mutations on complex I activity and ATP production [1,2,22,33]. These LHON models also show increased oxidative stress and heightened sensitivity to mitochondrial toxins, reinforcing the hypothesis that antioxidant compounds could confer neuroprotection [2,27,33]. While creating a faithful in vivo model of LHON is challenging due to species differences in mtDNA, researchers have employed clever approaches, such as allotopic expression of mutant human ND4 in rodents, to induce LHON-like optic neuropathy for therapeutic trials [78]. Through models like these, it has become clear that a delicate interplay between mitochondria and other cellular organelles governs RGC survival: for instance, *Wfs1*-knockout mice (modeling Wolfram syndrome) exhibit not only optic atrophy but also ER stress markers and altered mitochondrial calcium handling in retinal neurons [11,60,61]. Overall, animal and cellular models have been invaluable in dissecting the molecular cascade from genotype to RGC degeneration. They confirm that perturbations in mitochondrial dynamics, metabolism, and quality control are sufficient to cause optic neuropathy, and they provide experimental platforms to evaluate potential therapies targeting these pathways (Table 2).

**Figure 2:**
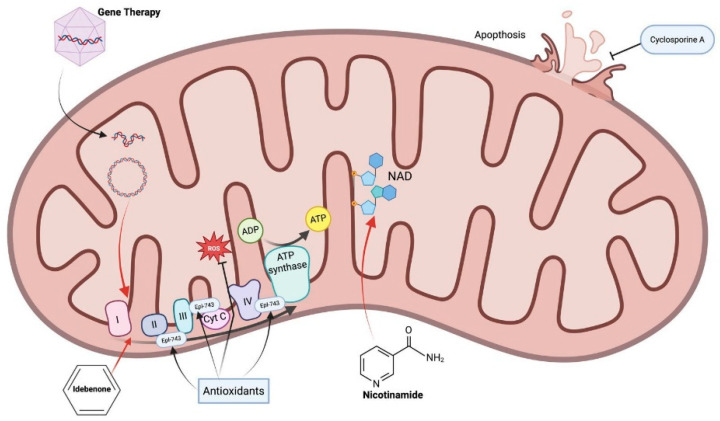
Different therapeutic approaches in hereditary optic neuropathies. Created in BioRender. Avitabile, A. (2025) https://BioRender.com/daxnqqg (accessed on 20 September 2025).

Encouragingly, recent advances in gene therapy are directly targeting the molecular defects in HONs. In LHON—long considered untreatable—gene therapy trials have achieved proof-of-concept success by employing adeno-associated virus (AAV) vectors to deliver functional copies of the mutated mitochondrial genes [26,79] (Table 3, Figure 2). Because mitochondrial DNA cannot be easily modified in situ, an “allotopic” gene strategy is used: the mtDNA gene (e.g., *MT-ND4* encoding NADH dehydrogenase subunit 4) is recoded and delivered to the nucleus via AAV, with added mitochondrial targeting sequences so the expressed protein is imported into mitochondria [26,79]. This approach has been tested in several clinical trials. A landmark phase 3 trial of AAV2-*ND4* (lenadogene nolparvovec) in LHON showed that a single intraocular injection led to clinically meaningful vision improvement in a subset of patients, and intriguingly even the contralateral sham-treated eyes showed some delayed improvement, suggesting inter-eye transfer of vector or protein [29]. More recently, bilateral administration of the same gene therapy was found to further improve visual outcomes compared with unilateral treatment, presumably by ensuring that both optic nerves receive the therapeutic gene [29]. These studies demonstrate that gene replacement can partially restore mitochondrial function in RGCs: treated patients have shown increased ganglion cell layer thickness and improved visual acuity relative to natural history [24,29]. Building on the *ND4* success, a gene therapy for the *MT-ND1* mutation (another cause of LHON) has entered clinical evaluation [80]. Parallel efforts are focusing on nuclear gene defects: because DOA results from *OPA1* haploinsufficiency, it is an ideal candidate for AAV-mediated gene augmentation. In a pivotal preclinical study, delivery of wild-type *OPA1* via AAV2 to the retina of Opa1-mutant mice preserved mitochondrial integrity and prevented optic nerve degeneration [53]. This demonstrates the feasibility of treating DOA at its molecular root by supplementing the missing *OPA1*. Beyond gene replacement, researchers are exploring genome editing technologies to correct pathogenic variants. In principle, CRISPR/Cas9-mediated editing could permanently correct a dominant negative *OPA1* mutation or knock out a toxic gain-of-function allele in vivo [81]. In fact, patient-derived cells with an *OPA1* missense mutation have been successfully “repaired” using CRISPR-Cas9 ex vivo, which restored normal mitochondrial network dynamics in those cells [55]. However, the application of CRISPR to HONs faces significant hurdles, especially for mtDNA lesions. The mitochondrial genome is not readily amenable to CRISPR editing due to the absence of efficient mechanisms to import guide RNAs into mitochondria and the lack of homologous recombination in that organelle [33,55]. To circumvent this, novel gene-editing tools are in development—for example, mitochondria-targeted base editors (which do not rely on guide RNA import) have shown promise in vitro for creating or correcting specific mtDNA mutations [33]. While not yet applied to LHON clinically, these tools could eventually allow direct fix of mtDNA mutations. For nuclear genes, AAV-based CRISPR delivery or base editing may offer a future one-time treatment for disorders like DOA, provided that off-target effects can be minimized and sufficient retinal coverage can be achieved [30,55]. In summary, gene therapy in HONs has advanced from theoretical to practical: AAV-mediated gene delivery is already yielding functional improvements in patients by addressing the underlying mitochondrial defects, and emerging gene editing technologies holds the potential for more precise, one-and-done corrections of HON mutations in the coming years [82,83,84,85].

Complementing gene-based therapies, several pharmacological approaches have been developed with a strong molecular rationale in HONs. The best-known example is idebenone, a synthetic short-chain benzoquinone and analog of coenzyme Q10. Idebenone can shuttle electrons directly from complex I to ubiquinone, bypassing partial blocks in complex I activity, and in doing so, it helps maintain ATP production in cells with defective complex I [26,27,28] (Table 2). This mechanism is highly relevant to LHON, where complex I dysfunction is the primary lesion [1,2,26,27]. By serving as an electron carrier and antioxidant, idebenone mitigates the energy failure and oxidative damage in RGCs harboring mtDNA mutations [2,26,27]. Clinically, idebenone has shown efficacy in improving or stabilizing vision, particularly when administered early in LHON onset [14,24,27,28]. The recent LEROS study reported that idebenone-treated LHON patients had better visual outcomes at 12 months compared with untreated controls [24], reinforcing the notion that bolstering mitochondrial electron transport can modify disease trajectory. There is also emerging evidence that idebenone may benefit other mitochondrial optic neuropathies; for instance, a small trial in DOA patients suggested slight improvements in visual acuity and color vision with high-dose idebenone, although larger studies are needed [52]. Another promising agent is elamipretide (also known as SS-31), a cell-permeable tetrapeptide that targets the inner mitochondrial membrane. Elamipretide selectively binds to cardiolipin, a unique phospholipid of the inner membrane, and stabilizes the structure of mitochondrial cristae and the electron transport chain complexes [86]. By preserving mitochondrial ultrastructure and reducing electron leak, elamipretide improves ATP synthesis efficiency and reduces ROS generation [86]. In animal models of mitochondrial disease, elamipretide has been shown to protect retinal and optic nerve integrity by maintaining mitochondrial function in neurons [86]. Although primarily investigated in systemic mitochondrial myopathies and heart failure, this drug’s mechanism—i.e., enhancing mitochondrial bioenergetics—is directly applicable to HONs [86]. Ongoing research is evaluating whether elamipretide can rescue RGCs in LHON models or patients by counteracting the mitochondrial membrane instability caused by complex I mutations [86]. Together, idebenone and elamipretide represent a new class of mitochondria-targeted therapeutics aiming to fortify RGCs against bioenergetic collapse. They illustrate how understanding the molecular defect (complex I dysfunction or inner membrane instability) leads to tailored treatments that bypass or compensate for that defect. Other neuroprotective strategies are also being explored, such as nicotinamide (Vitamin B3) supplementation to enhance mitochondrial NAD+/NADH balance and antioxidants like curcumin or resveratrol to reduce ROS—all grounded in the goal of preserving mitochondrial health in RGCs [49,86]. While these pharmacological agents alone may not cure HONs, they can delay RGC loss and extend the window for intervention, and they may synergize with gene therapies by creating a more hospitable cellular environment for RGC recovery [2,27,33,87].

## 5. Conclusions

Genetic optic neuropathies, syndromic and isolated, are a pleomorphic collection of disorders united by progressive optic nerve damage. Mitochondrial dysfunction seems to emerge as a core pathogenetic mechanism, but nuclear genes are implicated as well—particularly in mitochondrial function and cellular stress response. Over the past decade, improved genetic testing and imaging technology have significantly enhanced diagnostic accuracy, enabling earlier identification of those at risk. Still, treatment patterns go on to shift. Pharmacologic drugs such as idebenone are at least somewhat successful for some patients with LHON, and more absolute treatments in the form of novel gene therapies have been announced. To supply patient care most fully, physicians must integrate molecular data, clinical judgment, and supporting measures—at times, with counsel from neurologists, endocrinologists, and cardiologists. In the years to come, the success of these endeavors will rely on ongoing research into mitochondrial biology, new gene-editing platforms, and translational partnerships that advance benchside discoveries to the bedside. Furthermore, the wide phenotypic heterogeneity observed in hereditary optic neuropathies and inherited retinal dystrophies underscores the necessity for comprehensive molecular testing to facilitate early, accurate diagnosis and tailored therapeutic strategies. This study offers a comprehensive overview of the genetic, molecular, and clinical dimensions of inherited ocular neuropathies. The interplay of mitochondrial dysfunction, nuclear gene abnormalities, and environmental variables highlights the necessity for a multidisciplinary strategy in diagnosis and treatment. Progress in gene therapy, pharmacological neuroprotection, and molecular diagnostics presents novel avenues for individualized treatment. Future investigations should concentrate on biomarker identification and early intervention methodologies to enhance patient outcomes.

In summary, the molecular and mechanistic focus in HON research is yielding tangible progress on multiple fronts. Mitochondrial dysfunction has emerged not only as a unifying pathology in optic nerve degeneration but also as a tractable target for therapy. The genotype–phenotype complexity seen in disorders like LHON and DOA is increasingly explicable by considering mitochondrial genetics, dynamics, and cross-talk with the nucleus—reinforcing the need for comprehensive molecular profiling of patients. Advances in gene therapy demonstrate that even the long-standing challenge of treating mtDNA disorders can be overcome by creative molecular solutions, whereas gene editing and antisense approaches foreshadow a future of precise genetic rescue for both mitochondrial and nuclear HONs. Concurrently, mechanistic insights from models are directly informing treatments: for example, recognizing the role of oxidative stress in RGC death led to trials of antioxidants like idebenone, and understanding OPA1’s role in maintaining cristae encouraged the development of strategies to boost OPA1 expression or function. Going forward, continued research is needed to uncover additional modifiers (for instance, why certain mitochondrial haplotypes or yet-unidentified nuclear factors confer resistance to HON) and to determine how phenomena like mitophagy, mitochondrial transport in axons, and neuroinflammation contribute to RGC vulnerability. By deepening our molecular understanding of HONs, we can identify new therapeutic targets and optimize existing ones—moving closer to an era of truly mechanism-based, personalized treatments. Ultimately, a combination of approaches—gene replacement or editing to address the root genetic cause, plus mitochondria-targeted drugs to support neuronal survival—may prove most effective in preserving vision. Such a multipronged strategy, grounded in the mechanistic nuances of each patient’s disease, offers the best hope to halt or even reverse the optic neuropathies that have long been considered irreversible.

## Figures and Tables

**Table 2:** Major clinical trials (completed or ongoing) for hereditary optic neuropathies.

Trial (ID)	Intervention	Phase	Design	Population	Key Outcomes	Status
RHODOS (NCT00747487)	Idebenone 900 mg/day	II	RCT, placebo-controlled (24 wks)	LHON (<5 yrs onset, n = 85)	No significant primary outcome; secondary VA endpoints improved.	Completed (2011)
LEROS (NCT02774005)	Idebenone 900 mg/day (post-approval)	IV	Observational, uncontrolled	LHON (<5 yrs onset, n = 199)	Clinically relevant visual improvement compared with natural history.	Completed (2020)
RESCUE/REVERSE (NCT02652780)	Lenadogene nolparvovec (AAV2-ND4, unilateral)	III	Sham-controlled, double-masked RCT	LHON (ND4), n = 76	Bilateral visual improvement superior to natural history.	Completed (2020–21)
REFLECT (NCT03293524)	Lenadogene nolparvovec (bilateral injections)	III	Sham-controlled, double-masked RCT	LHON (ND4), n = 98	Significant bilateral VA improvement (+22 ETDRS letters).	Completed (2022)
Idebenone in DOA (no NCT ID)	Idebenone 900 mg/day (compassionate use)	II	Prospective open-label (12 months)	DOA (OPA1; n = 10)	Modest visual acuity improvement after 1 year.	Completed (2023)
NICODA (NCT06007391)	Nicotinamide (vitamin B3, oral)	II/III	Randomized pilot (12 months)	DOA/DOA+ (OPA1; adults > 18, n = 30)	Results pending; ongoing.	Recruiting (2025)
DAN-WS (NCT02829268)	Dantrolene sodium (oral)	Ib/IIa	Open-label dose escalation (6 months)	Wolfram syndrome (n = 24)	Well tolerated, no significant visual benefit.	Completed (2021)
TREATWOLFRAM (NCT03717909)	Sodium valproate vs. placebo (oral)	II	Double-blind placebo-controlled RCT	Wolfram syndrome (>5 yrs, n = 70)	Primary outcomes pending; ongoing.	Ongoing

**Table 3:** Molecular therapeutic targets and strategies for hereditary optic neuropathies.

Molecular Target	Therapeutic Strategy (Type)	Evidence (Stage)
Mitochondrial complex I (LHON mtDNA mutations, e.g., MT-ND4, ND1, ND6)	Idebenone (pharmacological antioxidant, bypassing complex I)	Clinical: EMA-approved; RHODOS trial showed visual acuity improvements in secondary endpoints.
Mitochondrial ND4 gene (m.11778G>A mutation in LHON)	Gene therapy (Allotopic expression)—lenadogene nolparvovec (AAV2-ND4)	Clinical: Significant visual acuity improvement in phase III trials (RESCUE, REVERSE, REFLECT).
OPA1 (haploinsufficiency) (DOA)	Gene therapy (gene augmentation)—AAV vector carrying wild-type OPA1	Preclinical: Effective RGC protection in DOA mouse models; no clinical trials yet.
OPA1 (haploinsufficiency) (DOA)	Antisense oligonucleotide (ASO)—TANGO technology to increase OPA1 expression	Preclinical: Effective in cell models; under investigation.
Mitochondrial biogenesis/NAD+ metabolism (energetic deficit in DOA, LHON)	Nicotinamide (Vitamin B3) (nutraceutical therapy)	Preclinical/Clinical: Improved mitochondrial function in cell models; ongoing phase II/III trials in DOA patients.
Retinal ganglion cell (RGC) neuroprotection	Stem cell therapy—intravitreal injection of autologous MSC	Clinical (early): SCOTS trial (phase I/II) reported visual improvements; further studies needed.
ER Ca^2^⁺ homeostasis (ER stress in Wolfram syndrome, WFS1)	Dantrolene sodium—ER stress reduction	Clinical: Phase Ib/IIa trial showed good tolerability; no significant vision improvement at 6 months.
ER stress/UPR response (WFS1, Wolfram syndrome)	Sodium valproate—ER stress modulation	Clinical: Ongoing phase II trial (TREATWOLFRAM) assessing long-term efficacy.

## Data Availability

The data cited in this study are available in PubMed at https://pubmed.ncbi.nlm.nih.gov.
