# Correction: Zeppieri et al. Isolated and Syndromic Genetic Optic Neuropathies: A Review of Genetic and Phenotypic Heterogeneity. *Int. J. Mol. Sci.* 2025, *26*, 3892

**DOI:** 10.3390/ijms262110784

**Published:** 2025-11-06

**Authors:** Marco Zeppieri, Caterina Gagliano, Marco Di Maita, Alessandro Avitabile, Giuseppe Gagliano, Edoardo Dammino, Daniele Tognetto, Maria Francesca Cordeiro, Fabiana D’Esposito

**Affiliations:** 1 Department of Ophthalmology, University Hospital of Udine, 33100 Udine, Italy; 2 Department of Medicine, Surgery and Health Sciences, University of Trieste, 34127 Trieste, Italy; 3 Department of Medicine and Surgery, “Kore” University of Enna, Piazza dell’Università, 94100 Enna, Italy; 4 Mediterranean Foundation “G.B. Morgagni”, Via Sant’Euplio, 95100 Catania, Italy; 5 Eye Clinic Catania University San Marco Hospital, Viale Carlo Azeglio Ciampi, 95121 Catania, Italy; 6 Imperial College Ophthalmic Research Group (ICORG) Unit, Imperial College, 153-173 Marylebone Rd, London NW1 5QH, UKf.desposito@imperial.ac.uk (F.D.); 7 Department of Neurosciences, Reproductive Sciences and Dentistry, University of Naples Federico II, Via Pansini 5, 80131 Napoli, Italy

## Error in Figure

In the original publication [1], there were copyright issues related to our Figures 1 and 2 as published in the previous version. Figure 1 was erroneously created without generating the “Confirmation of Publication and Licensing Rights” with BioRendering. Figure 2 was accidentally uploaded and used in our manuscript by our junior scientist. We were unaware that this previously published figure [2], was uploaded and unintentionally included in our published manuscript. We apologize to our colleagues for this inadvertent mistake. Figure 2 needs to be deleted from our paper because it belongs to the previously published paper: Amore, G.; Romagnoli, M.; Carbonelli, M.; Barboni, P.; Carelli, V.; La Morgia, C. Therapeutic Options in Hereditary Optic Neuropathies. *Drugs* **2021**, *81*, 57–86. https://doi.org/10.1007/s40265-020-01428-3. PMID: 33159657; PMCID: PMC7843467.

Two new figures have been generated to replace the previous ones in order to create “Confirmation of Publication and Licensing Rights” with BioRendering. These figures have been prepared as original graphic representations that have not been published in previous publications. Both updated original figures now have the proper licensing rights. The corrected Figure 1 and Figure 2 appear below. The scientific conclusions are unaffected. This correction was approved by the Academic Editor. The original publication has also been updated.

## Figures and Tables

**Figure 1:**
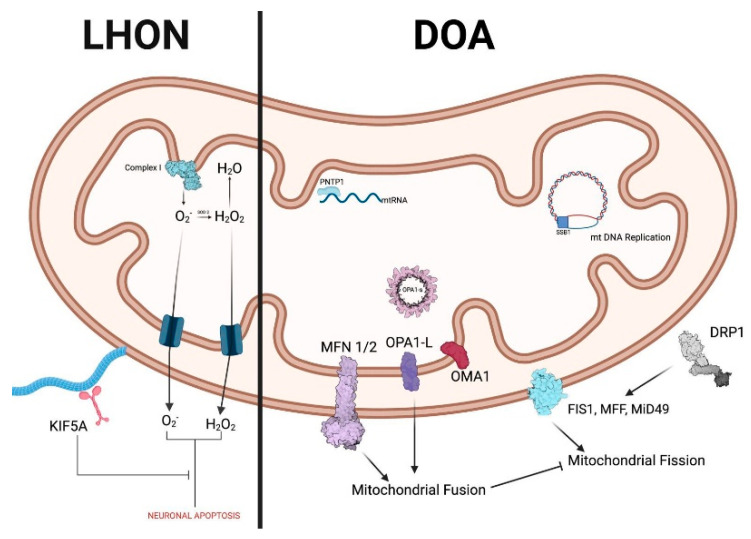
Summary of implicated proteins in LHON and DOA. Created in BioRender. Avitabile, A. (2025) https://BioRender.com/k6on15w (accessed on 6 September 2025).

**Figure 2:**
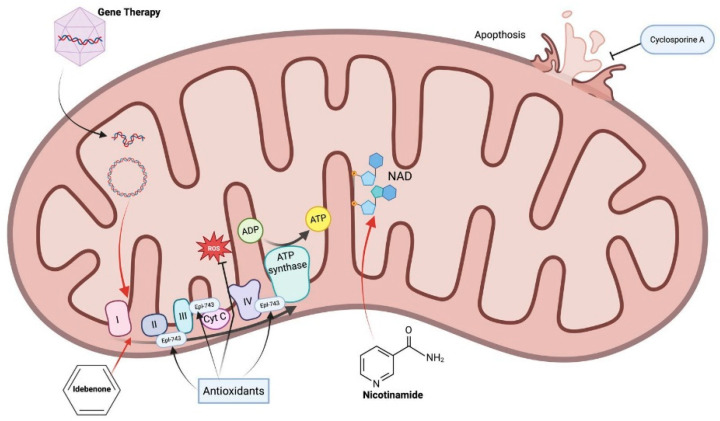
Different therapeutic approaches in hereditary optic neuropathies. Created in BioRender. Avitabile, A. (2025) https://BioRender.com/daxnqqg (accessed on 20 September 2025).
